# Magnetic Nanoparticles—A Multifunctional Potential Agent for Diagnosis and Therapy

**DOI:** 10.3390/cancers13092213

**Published:** 2021-05-05

**Authors:** Raghuraj Singh Chouhan, Milena Horvat, Jahangeer Ahmed, Norah Alhokbany, Saad M. Alshehri, Sonu Gandhi

**Affiliations:** 1 Department of Environmental Sciences, Jožef Stefan Institute, Jamova 39, 1000 Ljubljana, Slovenia; milena.horvat@ijs.si; 2 Department of Chemistry, College of Science, King Saud University, Riyadh 11451, Saudi Arabia; jahmed@ksu.edu.sa (J.A.); nhokbany@ksu.edu.sa (N.A.); 3 Amity Institute of Biotechnology, Amity University, Noida 201301, India; 4 DBT-National Institute of Animal Biotechnology (DBT-NIAB), Hyderabad 500032, India

**Keywords:** iron oxide nanoparticles, cancer, superparamagnetism, tumor microenvironment, magnetic nanoparticles

## Abstract

**Simple Summary:**

Magnetic nanoparticles (MNPs) are highly efficient contrast agents for magnetic resonance imaging for various types of tumors. This review focuses on the various approaches that can be used for selective targeting of MNPs towards a wide variety of solid tumors, with a specific emphasis on the surface modification that can affect the particles distribution in the tissue imparted by different targeting ligands for both in-vitro and in-vivo studies.

**Abstract:**

Magnetic nanoparticles gained considerable attention in last few years due to their remarkable properties. Superparamaganetism, non-toxicity, biocompatibility, chemical inertness, and environmental friendliness are some of the properties that make iron oxide nanoparticles (IONPs) an ideal choice for biomedical applications. Along with being easily tuneable and a tailored surface for conjugation of IONPs, their physio-chemical and biological properties can also be varied by modifying the basic parameters for synthesis that enhances the additional possibilities for designing novel magnetic nanomaterial for theranostic applications. This review highlights the synthesis, surface modification, and different applications of IONPs for diagnosis, imaging, and therapy. Furthermore, it also represents the recent report on the application of IONPs as enzyme mimetic compounds and a contrasting agent, and its significance in the field as an anticancer and antimicrobial agent.

## 1. Introduction

Early diagnosis and therapy of crucial diseases are pertinent for accurate diagnosis in a timely manner. Nanotechnology-based approaches paved the way for the diagnosis of various diseases. Improvement in the sensitivity and accuracy of diagnosis paved the way for efficient and early diagnosis. Various non-invasive in-vivo imaging techniques were developed for the diagnosis to visualize the progression of abnormal cells by binding to the target site. These non-invasive techniques include magnetic resonance imaging (MRI), single-photon emission computed tomography (SPECT), computed tomography (CT), nuclear imaging of positron emission tomography (PET), and optical (or fluorescence) imaging. The above said techniques are based on in-vivo imaging that assist the clinicians to locate the physiological and anatomical state of the organs.

IONPs majorly are used as a contrast agent in imaging, targeting, diagnostics, and treatment of hyperthermia. Magnetite (Fe_3_O_4_), maghemite (γ- Fe_2_O_3_) and hematite (α- Fe_2_O_3_) are some of the important examples of SPIONs [1]. The presence of Fe^2+^ and Fe^3+^ ions make SPIONs ideal candidate for biomedical applications. Superparamagnetic iron oxide nanoparticles (SPIONs) consisted of a ferrimagnetic group of the magnetic particles with major applications in biomedicine and bioengineering. Currently, SPIONs based therapeutics are at clinical, and preclinical trials, and few already reached to the market [2]. However, cellular toxicity occurred by SPIONs could potentially lead to adverse effects such as apoptosis, DNA damage, reactive oxygen species (ROS) generation, inflammation, mitochondrial function impairment, and chromosome condensation.

Polymeric materials such as polyethylene glycol (PEG), starch, and dextran polymers were widely used for the coating of SPIONs due to less toxicity, escaped by macrophages or spleen cells [3]. Advancement in the field of stimuli responsive polymers has occurred, due to a reversible phase transition upon exposure to environmental changes such as temperature, pH, light, enzyme, and the magnetic field [4]. The hydrophilic capsule of lipids due to its amphiphillic properties used for the coating of SPIONs includes various methods such as co-precipitation, thermal decomposition, sol-gel, microemulsion, electrochemical, and biosynthesis [5,6,7]. The present review aims to provide an insight into biomedical and clinical applications such as Magnetic Resonance Imaging (MRI), Magnetic Particle Imaging (MPI), Computed Tomography (CT), Positron Emission Tomography (PET), nanozyme applications for biosensing, gene and drug delivery, and hyperthermia and photothermal therapy, and broad spectrum antimicrobial applications in diagnosis of various diseases.

## 2. Biomedical and Clinical Applications

### 2.1. Magnetic Resonance Imaging (MRI)

MRI is an imaging technique that employs a strong magnetic field and radio waves for the creation of high-resolution images of various organs in the human and animal body. It has been used very widely in clinical radiology that uses non-ionizing radiation in experimental settings. Additionally, the modality is tomographic with the penetration of soft tissue with high resolution [4,8].

MRI utilizes the magnetic field, radio waves, or electric fields to illustrate the detailed internal structure of the body. MRI contrast agents can be divided into two categories, T1 and T2 agents. T1 agents alter the longitudinal relaxation time of water protons whereas T2 agents alter the transverse relaxation time of water protons. Positive T1—weighted, it shortens the T1 and has a moderate effect on T2 providing a brighter image. On the contrary negative contrast of T2/T2* weighted and shortens the T2 relaxation time and leads to the formation of dark images [9]. Tracking and monitoring of treatment, delivery of cells into the body, its proliferation, diffusion time, and migration rate require in-vivo imaging of the cells. Various imaging techniques are being used for this purpose, such as positron emission tomography (PET), single positron emission computed tomography (SPECT), X-ray based computed tomography (CT), and magnetic resonance imaging (MRI). Among all, MRI has shown to have a high spatial resolution up to 100 µm, in the absence of exposure to ionizing radiation [8], which makes it a great imaging technique for in-vivo cell imaging.

SPIONs are extensively used as contrast agents due to their superparamagnetic properties, biocompatibility, and low cost. SPIONs are used for visualizing tumor and metastatic cancer in liver, spleen, lymph nodes, and as a blood pool agent for angiography as well for inflammatory lesions. The nanoparticles reduce the relaxation time of the surrounding protons due to their superparamagnetic properties, which makes them suitable candidates as MRI contrast agents. IONPs based contrast agents were chosen over gadolinium-based contrast agents due to their low toxicity [10]. Ferucarbotran Resovist by Bayer Schering Pharma AG, contrast the effect of T1/T2, but most of their negative contrast and approved in few countries such as Japan, EU, and Australia [11]. Ferumoxytol (Feraheme) IONPs are approved by the FDA for the treatment of iron deficiency in patients suffering from chronic kidney diseases [12,13].

### 2.2. Magnetic Particle Imaging (MPI)

MPI is a tomographic technique that is non-invasive and detects tracers particles with superparamagnetic properties. MPI has potential applications in the field of diagnostics, imaging, and materials properties. MPI can be used to locate and measure the concentration of nanoparticles with non-ionizing radiation at any depth within the body to produce a signal. MPI signals are directly generated from the superparamagnetic nanoparticles when magnetized, which is 10^7^ times more sensitive than MRI signals. Magnetic particle spectrometer (MPS) has the potential for 3D in-vivo imaging with high special resolution where SPIONs were employed for lactoferrin conjugation. The MPS signal was recorded using a custom-built magnetic particle spectrometer (MPS) [14,15]. Overexpressed cancer cells secrete specific proteases, such as trypsin, matrix metalloprotease-2 (MMP-2), which was detected via MPS. Neutravidin-coated SPIONs were aggregated in the presence of biotin labeled peptides that were later selectively identified and cleaved by specific proteases which was present in the peptides (protease cleavage site) which led to the dispersion of the SPIONs. The aggregation or dispersion state of the SPIONs were identified by MPS signals through magnetic relaxation characteristics. Hence, MPS can be used in biomedical applications such as rapid detection of proteases in biological samples for example blood, urine, tissue extracts, and cell culture media for diagnosis of various types of cancer [16].

### 2.3. Computed Tomography (CT)

A computed tomography can be used in radiology for medical imaging to acquire detailed images of the body in a non-invasive manner. A CT scan measures X-ray attenuations of different tissues inside the body with rotating x-ray tube and detectors. The X-ray measurements usually taken multiple times from different angles to process on a computer to reconstruct the algorithms for the generation of tomographic images of a body. Contrast agents for CT are injected intravenously, the widely used agents are iodine conjugated small particles, and gadolinium-based contrast agents. However, patients with renal impairment are reported to be highly sensitive to iodine-based contrast agents, and gadolinium-based agents show toxicity in cell or tissues. Therefore, iron-oxide-based nanoparticles were taken into consideration as an alternate of iodine-based contrast agents [17,18].

Gold-coated iron oxide glycol nanoparticles can be used as an effective contrast agent for imaging in CT. It was confirmed that small size magnetic nanoparticles are highly biocompatible, biodegradable, and possessive of X-ray attenuation characteristics, and represent low toxicity over a longer period of time. These characteristics make them a strong candidate for both CT and MRI imaging [19,20]. Naha et al. synthesized a nanocomposite of bismuth-IONPs with dextran coating to check the cytotoxicity, accumulation, and half-life of the nanoconjugate. Lower cytotoxicity was observed in HepG2 (Human liver cancer cell line), and CT imaging was done after overnight incubation with nanoparticles to observe the contrast in blood vessels and heart. The nanoconjugate was found to have a protracted circulation half-life in the body. It was established that the nanoconjugate had a strong X-ray attenuation property and, biocompatibility that make it a good candidate as contrast agent in CT and MRI [19].

Reguera et al. designed and synthesized gold-iron-oxid- based Janus magnetic-plasmonic nanoparticles, which were used as a contrast agent for numerous techniques such as CT (Computed tomography), MRI (magnetic resonance imaging), TEM (transmission electron microscopy), PAI (photo acoustic imaging), SERS (surface enhanced Raman spectroscopy), optical imaging. Hence, by using these multifunctional nanoparticle agents, maximum cellular information can be obtained at once making them serve as great imaging tool in biomedical platform [21]. However, the adverse effects caused by the use of ionizing radiations sometimes restrict use in the patients.

### 2.4. PET (Positron Emission Tomography)

Positron emission tomography (PET) is an imaging technique that employs radioactive material known as radio-tracers to envision and measure changes in metabolic events. PET enables localization and quantification of activities in specific organs or tissues for whole body imaging; however, it cannot reveal about the anatomy or morphology of the tissues. Different tracers were used for imaging, based on the target such as 18F-FDG for cancer, NaF-F18 for bone formation, and oxygen-15 to measure blood flow. In PET, a radioisotope was attached to a drug, followed by injection into the body as a tracer. The emitted gamma rays were detected and used for the reconstruction of 3-D image. PET scanners can be combined with a CT scanner in the same session and are known as PET-CT scanners.

**Figure 1:**
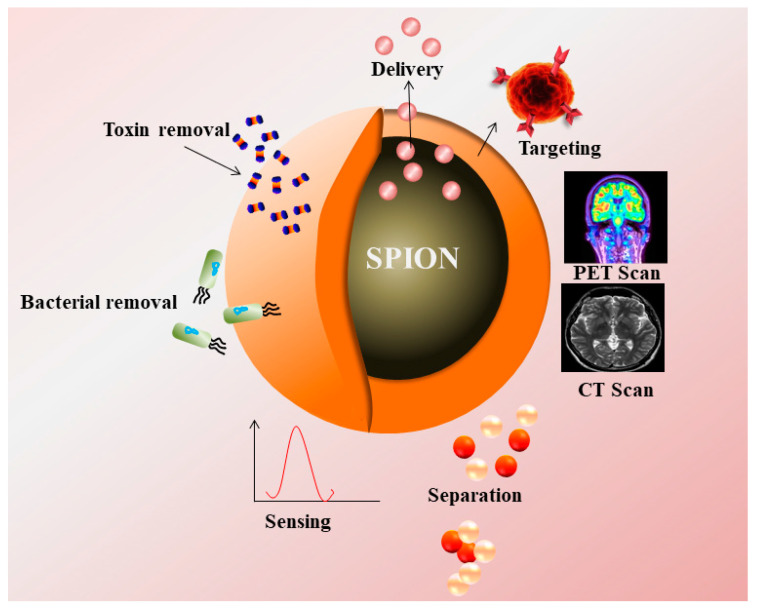
Superparamagnetic nanoparticles (SPIONs) as a contrast agent in PET, CT Scan, and their applications in targeting, drug delivery, removal of toxin, bacteria, and sensing.

Torres et al. developed a novel contrast agent composed of IONPs (Feridex) labeled with a ^64^Cu based bi-functional chelator for in-vivo imaging of lymph node [22]. Tri-modality agents were synthesized by conjugation of a PET tracer ^64^Cu with dextran coated magneto fluorescent nanoparticles for PET, MRI, and fluorescence imaging (Figure 1). Tri-modality nanoparticles were used to detect macrophages in atherosclerotic patients [23]. Stelter et al. functionalized IONPs with Ga-DTPA to synthesize a Ga-DTPA-IONP complex and injected into mouse model. PET and magnetic resonance imaging were performed to observe the accumulation of IONPs in kidney and spleen regions [24]. Glaus et al. synthesized IONPs micelles, which were radioactively labeled by ^64^Cu using DOTA as a chelating agent. In-vivo biodistribution was observed after 24 h of injection in mice through PET and MRI with 143 min half-life of the radioactive nanoconjugate, leading to accumulation in hepatic and splenic region [25]. Aryal et al. utilized ^64^Cu labeled PLGA functionalized SPIONs to locate breast cancer cells in a xenograft mouse tumor model. The nanohybrid was selected due to its longer circulation time as the nanohybrid was taken up by the tumor cells via EPR effect and located at the tumor region using PET and T2 weighed MRI [26]. Some SPIONs are approved by US-FDA for imaging applications in clinical settings (Table 1).

### 2.5. Nanozyme Applications for Biosensing

Nanozymes are the nanoparticles that exhibit the properties of enzymes such as oxidase, peroxidase, catalase, and superoxide dismutase. Various metals as well as metal oxides such as gold, silver, copper, SPIONs, and graphene nanosheets have shown HRP (horse radish peroxidase) like activities, which are currently being used for fabrication of biosensors. These non-enzymatic biosensors were used for detection of cholesterol concentration, creatinine, glucose, glutathione, H_2_O_2_, and urea. These biomolecules acted as biomarkers for various diseases, and biosensors developed for these molecules were used for early diagnosis of the diseases [38,39,40,41,42]. Studies showed that coating SPIONs with anionic SPIONs showed a higher affinity for TMB (3,3′,5,5′-tetramethylbenzidine), and cationic SPIONs for ABTS (2,2′-azino-bis (3-ethylbenzothiazoline-6-sulphonic acid) and OPD (O-phenylenediamine). Compared to natural enzymes, the nanozymes have various disadvantages such as slow substrate affinity and specificity. Therefore, novel methods for coating were used to enhance the catalytic activities of the nanozymes. Qian et al. synthesized nanozymes via conjugation of reduced graphene oxide on Fe_3_O_4_ nanospheres that was found to be stable for about three months when stored at 4 °C [43].

**Figure 2:**
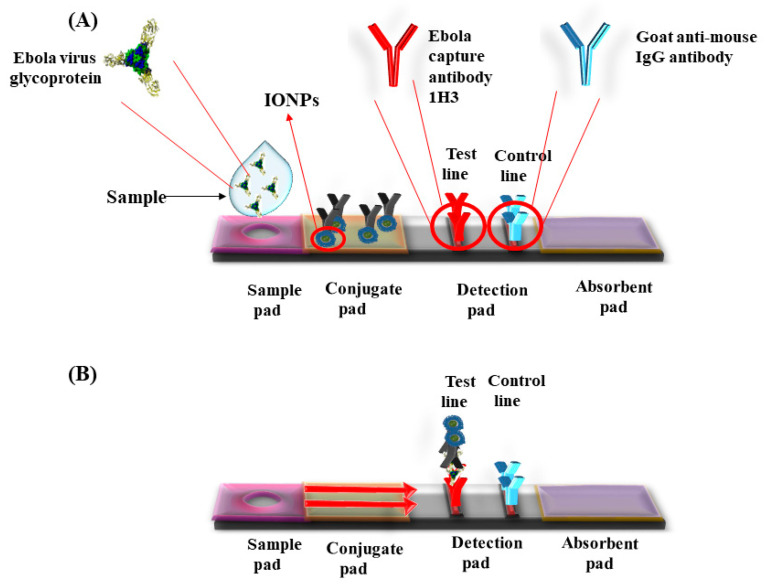
Lateral flow dipstick device for the detection of Ebola virus using Iron oxide nanoparticles (IONPs). (**A**) Ebola capture antibody 1H3 coated on test line and control line coated with goat anti-mouse IgG antibody. Sample was applied in the sample well. (**B**) The sample runs via capillary action on the membrane. Presence of specific antigen for Ebola developed color in the detection pad.

Super paramagnetic iron oxide nanoparticles (SPIONs) have catalytic properties of peroxides and catalase, which play an important role in preventing the cellular oxidative damage. Magnetite based iron oxide nanoparticles (IONPs) mimic the peroxidase activity and are used for fabrication of peroxidase based biosensors. Unlike the peroxidase enzyme, these IONPs do not degrade at unfavorable temperatures and pH (Table 2). Kacar et al. developed an amperometric sensor for creatinine using Fe_3_O_4_ nanoparticles that were modified with carbon paste electrodes. This method required two catalytic reactions, creatinase and sarcosine oxidase, to produce H_2_O_2_. The sensor detected the presence of H_2_O_2_ to determine the creatine level. The presence of elevated levels of creatine in urine or blood indicated the renal abnormalities or failure [44]. Gao et al. synthesized chitosan coated magnetic nanoparticles (CS-MNPs) for capture detection immunoassay, which detected the carcinoembryonic antigen (CEA) with a limit of detection (LOD) up to 1 ng/mL via sandwich ELISA, and direct ELISA [45]. Currently, various pathogens were detected by novel immunoassays such as IgG, *Mycoplasma pneumonia*, *Vibrio cholera*, Rotavirus, Hepatocellular Carcinoma biomarker, Golgi protein 73 (GP73) [46], Human Chorionic Gonadotropin (HCG), and cancer cells with Human Epidermal Growth Factor Receptor 2 (HER2) and Epidermal Growth Factor Receptor (EGFR) [47]. Peter et al. developed positively charged nanoparticles and monitored their localization in-situ by optical biosensor and transmission electron microscope (TEM). The positively charged nanoparticles penetrated well into the cells as compared with negatively charged particles with an optimal size around 5 nm for the cellular uptake [48]. A lateral flow assay was developed with IONPs’ nanozymes strip by Duan et al. for the detection of Ebola virus (EBOV). The nanostrip was capable to detect EBOV glycoprotein as low as 1 ng/mL (Figure 2). The diagnostic capability was compared with ELISA that required less time to detect EBOV [49]. 

## 3. Gene and Drug Delivery Using IONPs

### 3.1. Gene Delivery

**Figure 3:**
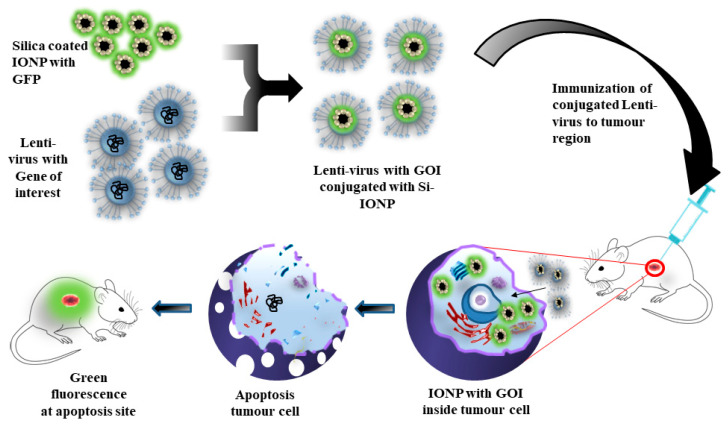
Silica-coated IONPs conjugated with lentiviral vector delivered the gene of interest at the targeted tumor region, and verified via green fluorescence, and cellular apoptosis at the tumor region.

Gene delivery is a technique for transfer of nucleic acids, siRNA, and plasmid DNA into targeted cells or tissues without being denatured by nucleases. Therefore, to prevent these biological materials from denaturation, nanocarriers such as IONPs were used to transport it to the target site in the body [55]. Borroni et al. created a novel vector for the transport of therapeutic genes for the treatment of a tumor. Silica-coated IONPs conjugated with a lentivirus vector was developed as a nanogene carrier to deliver the gene in the area of interest. Efficiency of the gene carrier was analyzed using a green fluorescence protein attached to IONPs and injected to a tumor bearing mice. It was observed that there was a presence of green fluorescence near the targeted tumor region (Figure 3) [56]. Cheong et al. synthesized IONPs nanocarriers and infused water soluble chitosan and linoleic acid (SCLNs,) which were used for targeting hepatocytes. The SCLNs were labeled with Technetium-99m, and nanoparticle localization was observed in the liver cells. SCLNs were further modified with GFP, administered intravenously, and GFP expression was observed in the primary hepatocytes for gene delivery and imaging [57].

Scherer et al. synthesized polyethyleneimine-coated magnetic nanoparticles and injected in-vitro without a viral vector for gene delivery. The major advantage of this technique was rapid sedimentation of magnetic particle-gene complex onto the targeted tumor region, which decreases the time as well as dosage of the particle gene complex to achieve efficient transfection. An association of DNA vectors with superparamagnetic nanoparticles increases the efficiency of transfection, and reduces the time of gene delivery to as low as 10 min [58].

Mahajan et al. developed a novel IONPs carrier to inhibit the progression of pancreatic cancer. IONPs nanocarriers were loaded with siRNA (siPLK1) directed against cell cycle specific serine-threonine-kinase (Polo-like kinase 1). siPLK1-IONPs were conjugated with myristolated polyarginine peptide (MPAP), a type of membrane translocation peptide that facilitated the transfer into the cytoplasm, and tumor-selective peptide under the glycosylated MUC1-specific peptide (EPPT1) that enhanced the tumor-specific delivery. It was observed that there was accumulation of nanoparticles, and an effective PLK1 silencing was observed, which led to tumor suppression via apoptosis [59].

### 3.2. Drug Delivery

Nanoparticles interact with cells, membranes, proteins, DNA, and organelles to form a series of nanoparticle-biological interfaces. The information about this phenomenon is required for safe use of nanomaterials [60]. The major application of iron oxide nanoparticles (IONPs) is the controlled rate of delivery of chemotherapeutic agents in targeted cells. Nanoparticles, when interacted with cells, showed less toxicity and targeting abilities. Margo et al. immobilized curcumin, a potential chemotherapeutic agent in surface active maghemite nanoparticles (SAMNs). The therapeutic properties of curcumin were preserved as well as there was a decrease in rapid oxidation and elimination of SAMNs [61].

Magnetic targeting studies were successful in many cases, but there are few reports in clinical trials to date. Phase I clinical trials were conducted for magnetic targeting, and delivery of drugs was done by Lubbe et al., where epirubicin was conjugated with the magnetic particles via electrostatic interactions between the phosphate group of the particles and amino group of sugars within the drugs. Two forms of treatment were being studied in mice and rats; with mechanical blockage of the tumor with a high concentration of ferro fluid and magnetic-particle-based targeted delivery of epirubicin with minimal particle concentration, no LD_50_ was found for the particles in the study. A clinical trial was performed on over 14 patients, and it was observed that epirubicin effectively targeted the tumor site. The nanoparticles were found to be accumulated in the liver but did not possess any anomalous effects [62,63,64]. Wilson et al. performed another clinical trial on 32 patients affected with hepatocellular carcinoma where doxorubicin hydrochloride conjugated with a magnetic nanocarrier, which was delivered into the body via hepatic artery catheterization. The nanoparticle-conjugated drug complex was targeted to the tumor area with the presence of an external magnetic field (500 mT), and the localization of the particles observed with MRI. Out of 32 patients, tumor targeting occurred in 30 patients effectively. In 15 patients, the tumor became stable or reduced in size, while in five patients, tumor progression was observed [65]. A similar study was done to measure the effectiveness of magnetic targeting of doxorubicin-loaded nanocarriers in four patients with hepatocellular carcinoma via a hepatic artery with MRI. The targeting of the particles towards the tumor region was done by placing rare earth magnets on the body. The results concluded that the particle-drug complex was well accumulated on the tumor sites, and 64–91% of tumor volume was affected by the particle-drug complex.

Cisplatin is a platinum-based anticancer agent used for various types of cancer such as lung, bladder, testicular, ovarian, and brain tumors [66]. Prolonged dosage of cisplatin was reported to cause various side effect such as kidney damage, allergic reactions, neurotoxicity, heart diseases, and bone marrow suppression [67], which reduced its chance of success in clinical trial. Yan Zhang et al. synthesized a novel nanoparticle with a Fe_3_O_4_ core, and the polymer inner shell was covered with PEG and folate group while cisplatin encapsulated in the inner shell. The kinetics of the in-vitro release of cisplatin was recorded at various pH, and it was observed that the acidic pH cytotoxic response occurred in HeLa cells. The folate group in the nanoconjugate targeted the folate receptors in HeLa cells [68].

Commercially available SPIONs are administered intravenously as the primary route of choice due to MRI contrast agents. The major challenge is the specific uptake by the target cells to ensure 100% systemic bioavailability. As SPIONs or IONPs binds non-specifically by plasma proteins, uptake occurs by reticuloendothelial system. However, it is achievable to increase the uptake of SPIONs in target tissue with novel surface functionalization methods and attachment of appropriate targeting ligands, along with magnetic focusing (Table 3).

## 4. IONPs for Hyperthermia and Photothermal Therapy

Hyperthermia is a therapeutic technique where heat is produced near a tumor region via energy source such as radiowaves, microwaves, ultrasound energy, and magnetism. Hyperthermia tumor therapy is mostly applied to surface tissues because cancer cells have poor cellular architecture and are more sensitive to heat than normal cells and tissues [75]. Nanoparticles mediated by hyperthermia have various advantages; they can be synthesized in colloidal suspensions and injected into the body for tracing using various targeting techniques. Nanoparticles can be synthesized in various sizes to cross the biological barriers so that they can reach the targeted region, cells or, tissues and generate heat.

Magnetic hyperthermia already reached the clinical trials (Nanotherm) [76] due to certain advantages for instance, chances of multiple hyperthermia cycles, as well as unlimited tissue penetration. IONPs possessed the properties as an MRI contrast agent, and were located inside the body via non-invasive techniques [77]. The drawback of magnetic hyperthermia is the truncated yield of heat from IONPs, which required a large number of nanoparticles at the targeted location to obtain the therapeutic temperature rise. The efficiency of heating produced by the particles diminishes as IONPs taken up by the cancer cells via endocytosis; as the aggregation reduces the Brownian motion of the particles, distribution of the particles also decreases the heating effect, which is a concentration dependent phenomenon also known as “thermal bystander effect” [78].

**Figure 4:**
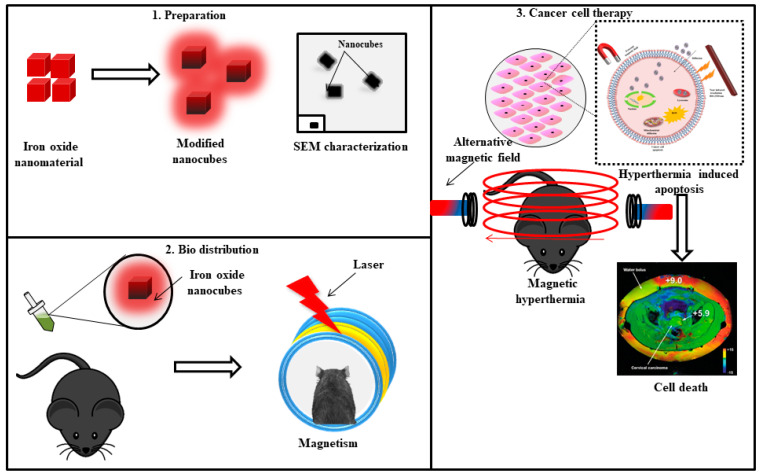
Iron oxide nanocubes for targeting cancer cells and ROS generation due to hyperthermia in the presence of external magnetic field and near infrared region (NIR).

IONPs can be used to synthesize nanohybrids, capable of magnetic hyperthermia and photothermia. Photothermal therapy can also be produced by other nanoparticles of gold, copper, silver, graphene, and carbon nanotubes [79,80,81,82]. Espinosa et al. synthesized iron oxide nanocubes when exposed to a magnetic field as well as near infrared irradiation (NIR); (Figure 4) the production of heat was amplified when compared to exposure only to the magnetic field; as well, dual mode stimulation generated higher heat irradiation with low IONPs concentration that is 0.25 M that leads to complete cell death in the tumor region [83].

Shen et al. used an iron oxide nanocluster for photothermal therapy (PTT) using near infrared region (NIR). It was observed that heat production amplified after radiation of the Fe_3_O_4_ nanocluster, thereby causing cytotoxicity to A549 cells (human lung cancer cells). The cell death mechanism was apoptosis rather than necrosis. Further in-vivo studies were performed to confirm the application of iron oxide nanoclusters in PTT [84].

Multifunctional IONPs were shown to provide better results than unifunctional IONPs. Zhou et al. developed PEGylated Fe_3_O_4_ nanoparticles to increase the bioavailability in the cells with three functions including PTT, MRI, and targeting. The nanoparticles targeted the tumor site (HeLa cells) in the presence of an external magnetic field, using a neodymium magnet where it exhibited the MRI signal, and photothermal therapy [85].

The major limitation of SPIONs for effective therapy is the insufficient magnetic gradient (distance between the target location and magnet) to control its residency period at the targeted site. As per the investigations, magnetite-based approaches must be in the range of 0.2 T with an approximate gradient of 8 T m^−1^ flux density at the target site That indicates that if the target site is close to the magnet source, more effective targeting will occur in the region with slow blood flow [86]. Table 4 summarizes the IONPs used in for magnetic thermal therapy/photo thermal therapy in cancer.

## 5. IONPs for Broad Spectrum Antimicrobial Applications

Photothermal therapy, magnetic resonance imaging (MRI), chemotherapy, and drug delivery are some of the prominent applications of IONPs. However, these nanoparticles also exhibit antimicrobial properties. Bacterial infections are among the most important infectious diseases, and special attention must be given to antibacterial agents due to the development of multi-drug resistance [95,96]. In recent days, antimicrobial resistance (AMR) is one of the main concerns, and therefore there is a constant need of an alternative treatment of microbial diseases. IONPs are emerging as an alternative, as microbes are unable to develop resistance against the inorganic elements [95]. Nanoparticles are emerging as an anti-microbial agent due to the fact that microbes are unable to develop resistance against inorganic elements and can be used repeatedly. There are 17 types of bacterial species, out of which five types responded to IONPs such as *Bacillus subtilis, Bacillus cereus, Aeromonas hydrophila, Escherichia coli*, and *Staphylococcus aureus*. Chemical conjugation of IONPs lead to enhanced effects against the microbes; citrate-coated IONPs inhibited the growth of certain species such as *Escherichia coli, Bacillus subtilis, Candida albicans, Aspergillus niger*, and *Fusarium solani* [97,98]. Rafi et al. synthesized 10 nm α-Fe_2_O_3_ nanoparticles via a thermal decomposition method that possessed antibacterial activity against three gram-positive bacterial species such as *S. aureus, A. hydrophila*, and *Streptococcus pyogenes*, and three gram-negative bacterial species such as *P. aeruginosa, E. faecalis*, and *E. coli* [99]. Zhang et al. treated *E. coli* cells with hematite for 45 min, and observed that hematite treated cells became stiffer than untreated cells [100].

Recently, green synthesis methods were applied for the synthesis of various nanoparticles. Fe_3_O_4_ nanoparticles were synthesized from the corn plant extract and were observed to have the antibacterial and anticandidal properties [101]. Another attempt was done by using cannon ball tree fruit extracts (*Couroupita guianensis*) to synthesize Fe_3_O_4_ NPs that showed bactericidal effects on various human pathogens [102]. Arokiyaraj et al. synthesized IONPs with *Argemone mexicana* leaf extract that inhibited the growth of *Escherichia coli* and *Proteus mirabilis* [103].

## 6. Conclusions and Future Perspectives

Super paramagnetic IONPs (SPIONs) and iron oxide nanoparticles (IONPs) are established as a T2 contrast agent, and are further applied in different biomedical applications such as MRI imaging, and as a probe for the detection of anomalies in brain, kidney, tissues, and organelles. Furthermore, with advancements, the iron oxide nanoparticles can be synthesized in various shapes and sizes, and can be functionalized with various biocomplexes with low toxicity towards the cells that make it a suitable candidate for drug and gene delivery. The predominant tumor cells seen to be affected by the IONPs are HEK293, A549, HeLa, and MCF-7. The risk of accumulation and tissue damage is reduced when compared to ordinary imaging methods; hence, nanoconjugates are recently being used for hyperthermia and photothermal therapy. It was observed that low concentrations of nanoconjugates are capable of enhancing heat generation at the target tumor region. Additionally, the nanoconjugates or nanohybrids showed enzyme mimetic properties that facilitated single step detection of biomolecules for nanosensors. The semi-conductive behavior of SPIONs is befitting and effective for antibacterial and antimicrobial applications, and since the microbes cannot develop any resistance for these complexes, it can be used for multiple duration.

Although considerable achievements have been made in the field of targeted delivery in cancers, how to prevent disturbing healthy cells remains unidentifiable. The combinatorial approach of nanoparticles in targeting ligands would lead to efficacious therapy. Therefore, more attention is required for the development of novel drug delivery mechanisms with multifunctional IONPs or SPIONs containing quantum dots (QD), polymers, and miRNA. The translational research in oncology depends on smarter carriers for delivery of drugs or tracers uncompromising the impairment to the healthy cells and tissues of the organs.

## Figures and Tables

**Table 1:** Clinically approved IONPs conjugated compounds and its applications at various biomedical platform.

Serial No.	Compound Name	Coating	Applications	Clinically Approved	References
1	Ferumoxtran (Combidex^®^)	Dextran	Lymph node imaging, macrophage imaging, blood pool agent, cell labelling, CNS imaging, MRI	In clinical trials	[27,28]
2	Ferucarbotran (Resovist^®^)	Carboxydextran	Liver imaging, cell labelling, CNS imaging, MRI	Approved	[29,30,31]
3	Ferumoxide (Feridex^®^)	Dextran	Liver imaging, cell labelling, CNS imaging, MRI	Withdrawn from market	[32,33,34]
4	Ferumoxytol (Feraheme^®^)	Carboxymethyl-dextran	Iron replacement therapy in patients with chronic kidney diseases	Approved	[35]
5	Feruglose (Clariscan™)	PEGylated starch	Blood pool agent, MRI	In clinical trials	[36]
6	Ferumoxsil (Gastromark^®^)	Siloxane	Oral GI imaging	Approved	[13,37]

**Table 2:** Chemically synthesized and modified IONPs nanozymes and its application for the detection of various biological compounds.

Serial No.	IONPs/Conjugated IONPs	Applications	References
1	Fe_3_O_4_ NPs	Exhibit peroxidase enzyme like activity. Used for fluorescent turn off system for detection of protein in urine	[50]
2	Chitosan coated IONPs with urease IONPs	Used for detection of urea	[51]
3	IONPs	Detection of *Brucella* antibodies with a LOD of 0.05 µg/mL	[52]
4	Fe_3_O_4_ nanocomposites/graphene oxide	Biosensor synthesis for glucose detection with the range 0.5–10 mM	[53]
5	Fe_3_O_4_ NPs loaded in Co_3_O_4_ nanocages	Used for glucose detection with the range of 0.5–30 µM with an LOD of 0.05 µM	[54]

**Table 3:** List of various iron oxide nanoparticles/chemically modified iron oxide nanoparticles used for loading anticancer drugs and gene for targeted delivery.

Serial No.	IONPs/Conjugated IONPs	Applications	References
1	IONP coated with doxorubicin (DOX) and 2-deoxy- D glucose	NPs when combined with doxorubicin and 2-deoxy-D-glucose showed enhanced chemotherapeutic actions in breast cancer cells via targeting	[69]
2	Dextran coated IONP conjugated with FITC and DOX	This nanoconjugate has various applications they are used for drug delivery, MRI, FITC fluorescence imaging, pancreatic cancer treatment via hyperthermia	[70]
3	Daunorubicin loaded IONPs	Used for treatment of brain glioma, it was observed that these NPs have the capacity to cross the blood brain barrier and act as a drug to treat blood cancer	[71]
4	DOX loaded in reduced graphene oxide coated IONPs	Caused inhibition of growth in HeLa cells when assisted with hyperthermia treatment	[72]
5	IONP conjugates with Homoharringtonine	Used for hematological anomalies, drug conjugated with IONPs were more effective in reducing tumor growth in case of leukemia in mice compared to only drug treatment	[73]
6	Fe_3_O_4_ nanoparticles	Used for tumor treatment using cryoablation therapy, extreme cold temperature is provided to destroy cells and tissues. Cryoprobes (thermally conductive fluids) are injected intravenously to the targeted regions	[74]

**Table 4:** Summary of functionalized IONPs with high potential for magnetic thermal therapy/photo thermal therapy in diminishing tumour affected cells.

Serial No	IONPs/Conjugated IONPs	Applications	References
1	Fe_3_O_4_/ICG/PFP encapsulated in PLGA	In vitro treatment of MCF-7 breast cancer cells via PTT, where the nanoconjugate is used an as agent for tumor termination	[87]
2	SPIONs	Hyperthermia based theraphy for liver cancer treatment	[88]
3	Carboxyl amine functionalized SPIONs	Terapthalic acid and amino terapthalic acid coated SPIONs caused in vitro hyperthermia and induces cell death in MCF-7 breast cancer cells	[89]
4	IONP functionalized with HAS protein	Used for magnetic thermal therapy where the MNPs at 36 °C produce a localized heat in presence of alternating magnetic field	[90]
5	CMCT functionalized Fe_3_O_4_ NPs	Used for photothermal therapy where the NPs found to be accumulated at the tumour region and due to PTT, there is an increase in temperature up to 52 °C	[91]
6	Fe_3_O_4_/NiFe_2_O_4_ NPs coated with oleic acid	Used for magnetic hyperthermia therapy where oleic acid coated NP clusters were targeted in vitro in HeLa cells and in presence of external magnetic field an increase in temperature was observed.	[92]
7	NanoTherm™ Aqueous dispersion of superparamagnetic iron oxide nanoparticles	Used for hyperthermia and currently in clinical trial phase	[76]
8	NCT01270139 Iron-bearing nanoparticles	Used for hyperthermia and currently in clinical trial phase	[93]
9	NCT01436123 Gold nanoparticles with iron oxide-silica shells	Used for hyperthermia and currently in clinical trial phase	[94]
